# Optical properties of orthodontic aligners—spectrophotometry analysis of three types before and after aging

**DOI:** 10.1186/s40510-015-0111-z

**Published:** 2015-11-18

**Authors:** Luca Lombardo, Angela Arreghini, Roberta Maccarrone, Anna Bianchi, Santo Scalia, Giuseppe Siciliani

**Affiliations:** Postgraduate School of Orthodontics, University of Ferrara, Via Montebello 31, 44100 Ferrara, Italy; Department of Chemical and Pharmaceutical Sciences, University of Ferrara, Via Fossato di Mortara 17/19, 44121 Ferrara, Italy

**Keywords:** Orthodontic aligners, Transparency, Spectrophotometry, F22 Aligner

## Abstract

**Background:**

The aim was to assess and compare absorbance and transmittance values of three types of clear orthodontic aligners before and after two cycles of in vitro aging.

**Methods:**

Nine samples of orthodontic aligners from three different manufacturers (*Invisalign*, Align Technology, Santa Clara, CA, USA; *All-In*, Micerium, Avegno, GE, Italy; *F22 Aligner*, Sweden & Martina, Due Carrare, PD, Italy) were selected, and each sample was subjected to spectrophotometry analysis of both its transmittance and absorbance a total of 27 times. Samples were subsequently aged in vitro at a constant temperature in artificial saliva supplemented with food colouring for two cycles of 14 days each. The spectrophotometry protocol was then repeated, and the resulting data were analysed and compared by means of ANOVA (*p* < 0.05).

**Results:**

All types of aligners tested yielded lower transmittance and higher absorbance values after aging, but the difference was not significant in any case. That being said, the F22 aligners were found to be most transparent, both before and after aging, followed by Invisalign and All-In, and these differences were statistically significant.

**Conclusions:**

Commercial aligners possess significantly different optical, and therefore aesthetic, properties, both as delivered and following aging.

## Background

The rising demand among adult patients for “invisible” orthodontic treatment has led to an exponential growth in the clear aligner market [1]. Indeed, these aligners have low aesthetic impact [2–4], as well as being able to effectively and progressively guide the teeth into their programmed positions. They are also removable and therefore do not hamper oral hygiene maintenance, in turn reducing the risk of white spots, caries, gingivitis and periodontal disease. Being manufactured sequentially to specification from digital malocclusion set-ups and delivered direct from the factory, they are also convenient for the orthodontist, who has to deal with none of the bracket- and archwire-related issues that plague fixed appliances [5].

Although clear aligners designed on manual set-ups had long been in use [6], it was only with the advent of Align Technology’s entirely digital Invisalign process—exploiting digital scans of patients’ models, CAD–CAM technology and 3D printing—that they became an accepted orthodontic technique [7–9]. Upon expiry of the Invisalign patent, a profusion of other aligner manufacturers sprang up, offering products based on different scanning technologies, set-up software, material, thickness, transparency and finishing quality and technique. In general, aligner materials are resin polymers, which, not being inert, are subject to changes in the warmth, humidity, mastication forces and prolonged contact with salivary enzymes in the oral environment [10]. The first aligners were made out of single-layer rigid polyurethane (from methylene diphenyl diisocyanate and 1,6-hexanediol) sheets. Although polyurethane is not inert, and is therefore subject to alteration in vivo, it does seem to be relatively stable in saliva, even though its hydrophilic tendencies will depend on the chemical bonds within it [11].

Nevertheless, Align Technology issued first Exceed-30 (EX30), a flexible material with improved transparency and fracture resistance, and then, in 2012, after 8 years of experimentation, a newly patented material called SmartTrack®. This is a thermoplastic polyurethane with an integrated elastomer that the firm maintains which is able to apply continuous light forces to the teeth and whose greater elasticity should guarantee greater predictability in terms of orthodontic movements [8–12]. At the present time, however, the other manufacturers make greater use of polyethylene terephthalate glycol (PeT-G) [13], followed by polypropylene (PP), polycarbonate (PC), thermoplastic polyurethanes (TPU), ethylene vinyl acetate (EVA) and many more. Aligner thickness tends to vary between 0.50 and 1.5 mm [14].

A relative newcomer to the market, the F22 Aligner, has been designed and manufactured by the Postgraduate School of Orthodontics at the University of Ferrara, Italy. Fruit of long years of experience in the field, it is made out of a chemically inert rigid polyurethane that possesses excellent properties such as transparency, ductility and resistance to stress [15, 16].

Although the mechanical (force release, rigidity, dimensional stability and wear) and chemical (stability) properties of such aligners have been extensively investigated [11, 17, 18], no scientific, reproducible studies into their transparency, i.e. the ability of light to pass through their constituent material, have yet been conducted [19]. Nevertheless, the transparency of these aligners is the major key to their success. Not all materials possess the same chemical characteristics, either before or after wear, and it therefore follows that the optical properties, alongside their mechanical properties, of different aligners will be affected differently by the masticatory stress, salivary enzymes, food colourings, etc. that they are exposed to in the oral environment during their 14 consecutive days of wear (at least 22 h per day, according to the manufacturers’ recommendations) [11]. Hence, we set out to compare the transmittance and absorbance of various samples of clear aligners, both as received and after two 14-day cycles of in vitro aging, to determine whether they present any differences in terms of colour stability and aesthetic properties over time.

## Methods

**Fig. 1:**
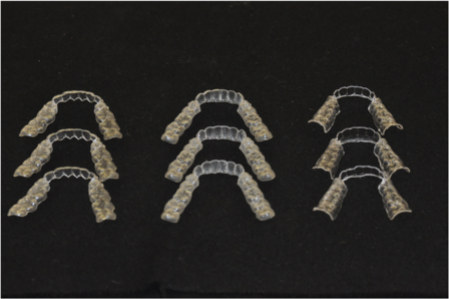
The nine aligner samples used in the experiment

Three identical samples of three different aligners from three different manufacturers were selected: three from Invisalign (Align Technology, Santa Clara, CA, USA), three from All-In (Micerium, Avegno, GE, Italy) and three from F22 (Sweden & Martina, Due Carrare, PD, Italy), designed for patients with well-aligned incisors and similar arch forms and dimensions (Fig. 1).

**Fig. 2:**
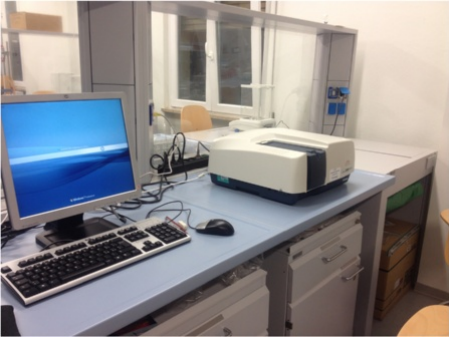
The UV–vis Jasco mod.V630PC spectrophotometer

The optical properties (absorbance and transmittance) of each sample of each aligner were assessed by means of spectrophotometry (Jasco UV–vis V630PC, Tokyo, Japan) (Fig. 2). The spectrophotometer in question has the following features: double beam, single monochromator, deuterium light source for UV range and halogen lamp for visible range, and a detector positioned 1 cm behind the detection window. The visible spectrum of light, i.e. of wavelength 400–700 nm, was considered, and the absorbance and transmittance of each sample were automatically recorded by the spectrophotometer. The *transmittance* is the fraction of incident light, at an established wavelength, that passes through the material; the greater the transmittance, the more transparent the material. Conversely, the greater the *absorbance*—the logarithmic inverse of the transmittance—the less transparent the material [20].

**Fig. 3:**
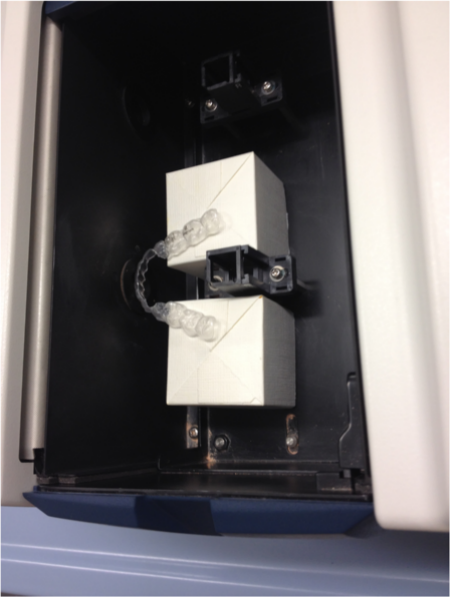
Positioning the samples in the spectrophotometer

Before these measurements were made, each aligner was sectioned from canine to canine with a rotating saw to remove the lingual portion and thereby expose the labial wall. Each aligner was cleaned by running under a jet of distilled water and dried with a jet of air. Each sample was then placed in a specially designed support, 35 mm in height, used to standardize the position of the aligner inside the spectrophotometer with respect to the detection window. The aligners were positioned so that the labial surface of the lower incisors was vertical and in contact with the incident light collection window (Fig. 3). Each sample was measured three times consecutively, varying the inclination of the aligner with respect to the light beam slightly, giving a total of 27 absorbance measurements and 27 transmittance measurements. The spectrophotometer was calibrated by means of the white light spectrum before each measurement.

The values yielded by the three aligner types as received were then compared via Spectra Manager II software (Jasco, Tokyo, Japan), which was used to plot an average curve (±SD) of the nine measurements made for each type.

**Fig. 4:**
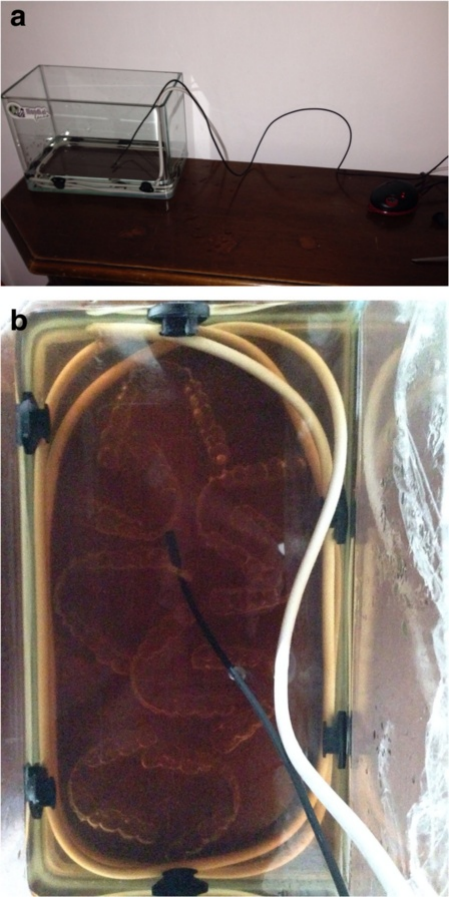
**a** Saliva bath used to age the samples. **b** Aging protocol

The same nine aligners were then immersed in a glass container measuring 30 cm × 17 cm × 14 cm filled with Oral Balance artificial saliva (Biotène Oral Balance, Biopharm Sas, Peschiera Borromeo, Italy), i.e. 250 ml of gel diluted in 1 L of water, to which brown food colouring was added in a 1:1 ratio. The saliva bath was kept at a constant temperature via an immersion heat source connected to a thermostat set at 37 °C ± 1 °C, and the aligners were left in situ for 14 consecutive days. As per previously published aging protocols (Fig. 4a, b) [11], this procedure was repeated, this time with yellow rather than brown food colouring, thereby subjecting the aligners to a total of two 14-day aging cycles.

After aging, the aligners were rinsed with distilled water, dried with an air jet and then subjected once again to the above-described spectrophotometry protocol. Transmittance and absorbance curves generated before and after aging were compared, and ANOVA (Graph Pad) was used to statistically analyse the data (*p* < 0.05).

## Results

**Fig. 5:**
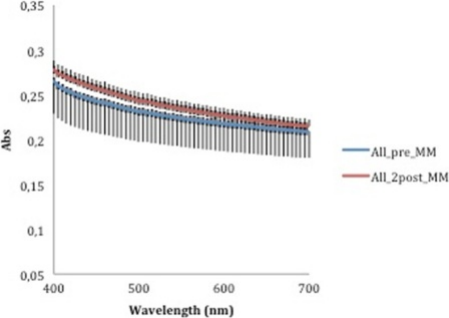
Comparison of absorbance values of the All-In aligner before and after aging. The *curves* are the average of nine spectrophotometry measurements ± SD

**Fig. 6:**
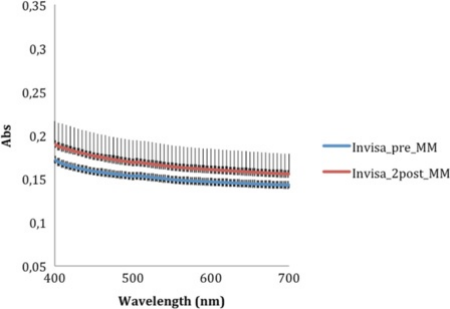
Comparison of absorbance values of the Invisalign aligner before and after aging. The *curves* are the average of nine spectrophotometry measurements ± SD

**Fig. 7:**
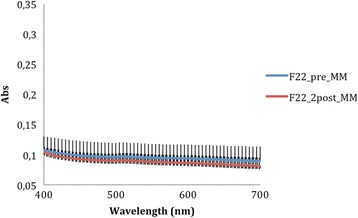
Comparison of absorbance values of the F22 aligner before and after aging. The *curves* are the average of nine spectrophotometry measurements ± SD

The absorbance data recorded by the spectrophotometer (Tables 1 and 2) at 400 nm and 700 nm—selected as the limits of the measurement range—were used to generate average curves for each aligner type, before and after aging. The absorbance curves of the All-In aligners before and after aging (Fig. 5) are both homogeneous, with mean values at wavelength 400 and 700 nm, respectively, of 0.265 ± 0.036 and 0.209 ± 0.030 for the former and 0.278 ± 0.011 and 0.215 ± 0.008 for the latter. Similarly, homogeneous absorbance curves were obtained for the Invisalign appliances (Fig. 6), but the mean absorbance at 400 nm and 700 nm were, respectively, 0.172 ± 0.004 and 0.143 ± 0.004 as supplied and 0.190 ± 0.027 and 0.155 ± 0.023 after aging. The before and after aging absorbance curves yielded by the F22 appliance (Fig. 7) were also homogeneous and almost superimposable. Indeed, before aging, the absorbance values at 400 and 700 nm, respectively, were 0.107 ± 0.023 and 0.090 ± 0.024 falling little after two aging cycles to 0.104 ± 0.003 and 0.081 ± 0.004.

**Table 1:** Absorbance values of the three aligners before and after in vitro aging

Aligner	Absorbance at 400 nm (±SD)			Absorbance at 700 nm (±SD)		
	As received	After aging	Significance	As received	After aging	Significance
All-In	0.265 ± 0.036	0.278 ± 0.011	N.S.	0.209 ± 0.030	0.215 ± 0.008	N.S.
Invisalign	0.172 ± 0.004	0.190 ± 0.03	N.S.	0.143 ± 0.004	0.155 ± 0.023	N.S.
F22	0.107 ± 0.023	0.104 ± 0.003	N.S.	0.090 ± 0.024	0.081 ± 0.004	N.S.

*N.S.* not significant variation

**Table 2:** Transmittance values of the three aligners before and after in vitro aging

Aligner	Transmittance at 400 nm ± SD			Transmittance at 700 nm ± SD		
	As received	After aging	Significance	As received	After aging	Significance
All-In	53.58 ± 4.1	51.08 ± 2.6	N.S.	60.72 ± 2.9	59.60 ± 2.6	N.S.
Invisalign	68.65 ± 1.1	63.38 ± 3.0	N.S.	72.66 ± 1.1	69.27 ± 2.4	N.S.
F22	79.29 ± 5.0	78.25 ± 1.2	N.S.	82.45 ± 4.6	82.45 ± 1.7	N.S.

*N.S.* not significant variation

**Fig. 8:**
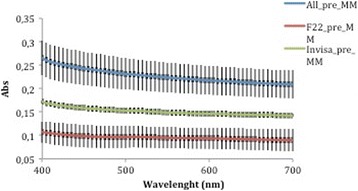
Absorbance curves ± SD of the three aligners before aging

**Fig. 9:**
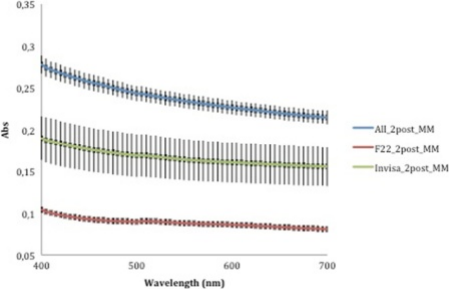
Absorbance curves ± SD of the three aligners after aging

Comparison of the three curves generated before aging (Fig. 8) reveals that the F22 absorbance values were lower than those produced by Invisalign and that All-In values were always the highest recorded. According to ANOVA, these differences were significant at all wavelengths of visible light (Table 3). The same trend was seen after aging (Fig. 9), and in this case too the differences between aligners were significant (Table 4).

**Table 3:** Pre-aging absorbance values of the three aligners

Wavelength (nm)	All-In	Invisalign	F22	*p* value
400	0.265 ± 0.036	0.172 ± 0.004	0.107 ± 0.023	<0.0007
450	0.242 ± 0.035	0.158 ± 0.003	0.098 ± 0.024	<0.0012
500	0.230 ± 0.033	0.152 ± 0.003	0.096 ± 0.023	<0.0012
550	0.223 ± 0.031	0.148 ± 0.003	0.094 ± 0.022	<0.0013
600	0.217 ± 0.030	0.146 ± 0.003	0.093 ± 0.022	<0.0012
650	0.212 ± 0.030	0.144 ± 0.003	0.091 ± 0.023	<0.0015
700	0.209 ± 0.030	0.143 ± 0.004	0.090 ± 0.024	<0.0015

Significance level *p* < 0.05

**Table 4:** Post-aging absorbance values of the three aligners

Wavelength (nm)	All-In	Invisalign	F22	*p* value
400	0.278 ± 0.011	0.190 ± 0.03	0.104 ± 0.003	<0.001
450	0.257 ± 0.010	0.176 ± 0.03	0.092 ± 0.003	<0.001
500	0.243 ± 0.009	0.168 ± 0.03	0.089 ± 0.003	<0.001
550	0.234 ± 0.009	0.163 ± 0.02	0.087 ± 0.003	<0.001
600	0.226 ± 0.008	0.160 ± 0.02	0.086 ± 0.003	<0.001
650	0.219 ± 0.008	0.157 ± 0.02	0.083 ± 0.004	<0.001
700	0.214 ± 0.008	0.155 ± 0.02	0.080 ± 0.004	<0.001

Significance level *p* < 0.05

**Fig. 10:**
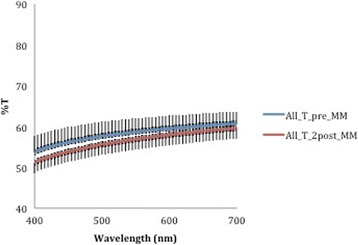
Comparison of transmittance values of the All-In aligner before and after aging. The *curves* are the average of nine spectrophotometry measurements ± SD

**Fig. 11:**
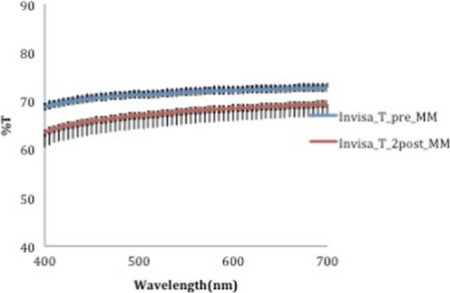
Comparison of transmittance values of the Invisalign aligner before and after aging. The *curves* are the average of nine spectrophotometry measurements ± SD

**Fig. 12:**
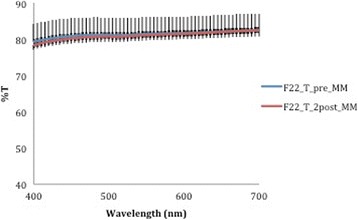
Comparison of absorbance values of the F22 aligner before and after aging. The *curves* are the average of nine spectrophotometry measurements ± SD

**Fig. 13:**
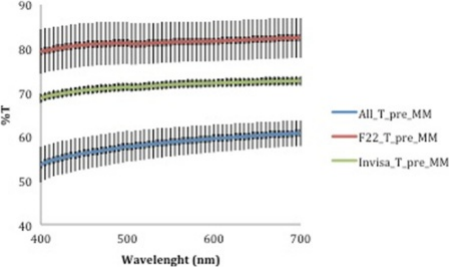
Transmittance curves ± SD of the three aligners before aging

**Fig. 14:**
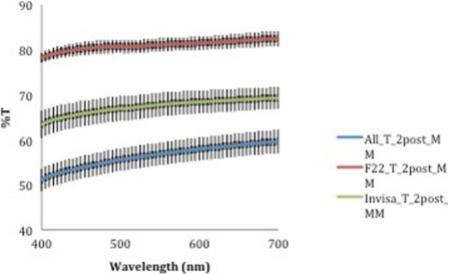
Transmittance curves ± SD of the three aligners after aging

**Fig. 15:**
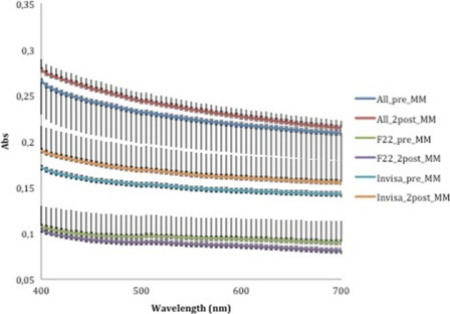
Comparison of absorbance values of the three aligners before and after aging

**Fig. 16:**
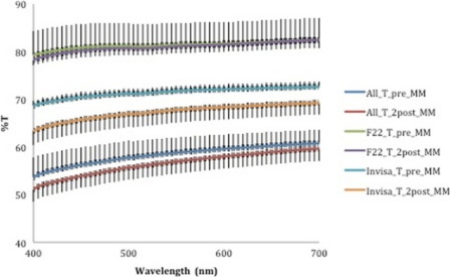
Comparison of transmittance values of the three aligners before and after aging

A corresponding inverse pattern was seen in transmittance values (Figs. 10, 11, 12, 13 and 14) and, once again, the F22 aligner provided significantly better optical properties (higher transmittance) at all wavelengths than its competitors (Tables 5 and 6). Differences between each aligner type in absorbance and transmittance before and after aging (Tables 1 and 2) were not, however, significant, although it is interesting to note that the smallest percentage variations in optical properties (Table 7) were recorded for the F22 aligners, indicating their greater stability under these experimental conditions (Figs. 15 and 16).

**Table 5:** Pre-aging transmittance values of the three aligners

Wavelength (nm)	All-In	Invisalign	F22	*p* value
400	53.58 ± 4.1	68.65 ± 1.1	79.29 ± 5.0	<0.0005
450	56.20 ± 3.7	70.51 ± 1.0	80.82 ± 5.0	<0.0005
500	57.74 ± 3.5	71.29 ± 1.0	81.21 ± 4.7	<0.0005
550	58.73 ± 3.3	71.80 ± 1.0	81.43 ± 4.6	<0.0005
600	59.55 ± 3.1	72.16 ± 1.0	81.67 ± 4.5	<0.0005
650	60.21 ± 3.0	72.44 ± 1.1	82.08 ± 4.6	<0.0005
700	60.72 ± 2.9	72.66 ± 1.1	82.45 ± 4.6	<0.0005

Significance level *p* < 0.05

**Table 6:** Post-aging transmittance values of the three aligners

Wavelength (nm)	All-In	Invisalign	F22	*p* value
400	51.08 ± 2.6	63.38 ± 3.0	78.25 ± 1.2	<0.001
450	53.84 ± 2.5	65.74 ± 2.8	80.21 ± 1.5	<0.001
500	55.64 ± 2.5	66.96 ± 2.8	80.76 ± 1.5	<0.001
550	56.87 ± 2.6	67.80 ± 2.7	81.10 ± 1.5	<0.001
600	57.94 ± 2.6	68.39 ± 2.6	81.45 ± 1.5	<0.001
650	58.85 ± 2.6	68.87 ± 2.5	81.97 ± 1.6	<0.001
700	59.60 ± 2.6	69.27 ± 2.4	82.45 ± 1.7	<0.001

Significance level *p* < 0.05

**Table 7:** Percentage variations in aligner absorbance and transmittance after in vitro aging

	% variation at 400 nm		% variation at 700 nm	
Aligner	Absorbance	Transmittance	Absorbance	Transmittance
All-In	+5.77	−4.53	+3.86	−1.83
F22	−0.74	−1.09	−6.55	+0.14
Invisalign	+10.46	−7.70	+8.73	−4.68

## Discussion

To our knowledge, only one previous study has investigated aligner transparency using spectrophotometry, but in that case only the absorbance of one brand (Invisalign) was measured [11]. Our spectrophotometry results showed that, of the three types tested, F22 had significantly lower absorbance and significantly higher transmittance than All-In and Invisalign, respectively. The difference in optical properties between the three brands was found both as received and after aging, indicating that the F22 aligners remain more transparent throughout treatment, although the chromatic stability of all sample types deteriorated during the aging process. This is in line with findings by Gracco et al. that both in vitro aging (artificial saliva) and in vivo wear increase the absorbance after 14 days. We show that this variation in absorbance, and, inversely, transmittance, was more pronounced with the All-In and Invisalign aligners (even more so) with respect to the F22 appliance. However, the differences between before and after aging measurements were not significant in any case, and we can therefore state that all three aligners tested maintain their optical properties in the presence of artificial saliva for at least 14 days (the recommended period of use). That being said, being tested in vitro, our samples were not subjected to any additional chemical (e.g. acidic drinks and oral enzymes) or mechanical stress (e.g. chewing, bruxism, removal and reinsertion), so we are unable to state to what extent their optical performance would change in vivo. Indeed, other studies [11] tell us that the optical properties of aligners deteriorate still further when worn for the same period in the mouth [21–23].

Another study has previously attempted to accelerate the aging process by means of a more aggressive treatment (a solution of 75 % ethanol and 25 % water for 2 weeks at 23 °C), but even this was unable to truly replicate the oral conditions [24]. Hence, we elected to age our samples in artificial saliva, the focus being on standardizing the stress to which the aligners were exposed and therefore the changes observed. Nevertheless, only a similar study conducted in vivo will confirm whether or not the differences in transparency between the aligners are also significant after 2 weeks in the oral environment.

## Conclusions

The optical properties of orthodontic aligners appear to vary between brands and constituent materials but deteriorate with in vitro aging in all cases. Of those tested, both before and after aging, F22 was significantly more transparent than All-In and Invisalign, whose optical properties were most changed by the process. Further studies will be required to measure the absorbance and transmittance of aligners after a cycle of wear in vivo, as in vitro testing conditions are unable to accurately reproduce the conditions in the oral environment.

## References

[1] Rosvall MD, Field HW, Ziuchkovski J, Rosenstiel SF, Johnston WM (2009). Attractiveness, acceptability, and value of orthodontic appliances. Am J Orthod Dentofacial Orthop.

[2] Boyd R, Miller RJ, Vlaskalic V (2000). The Invisalign system in adult orthodontics: mild crowding and space closure cases. J Clin Orthod.

[3] Kim TW, Echarri P (2007). Clear aligner: an efficient, esthetic, and comfortable option for an adult patient. World J Orthod.

[4] Kohda N, Iijima M, Muguruma T, Brantley WA, Ahluwalia KS, Mizoguchi I (2013). Effects of mechanical properties of thermoplastic materials on the initial force of thermoplastic appliances. Angle Orthod.

[5] Lingenbrink JC, King G, Bollen AM, Hujoel P, Huang G, Orsini-Alcalde G (2002). Quality of life comparison between clear removable and conventional orthodontics. J Dent Res.

[6] Kesling H (1945). The philosophy of the tooth positioning appliance. Am J Orthod.

[7] Align Technology Inc (2002). The Invisalign reference guide.

[8] Align Technology Inc. Material safety data sheet. MSDS Aligner EX203040 Customer support. Santa Clara: Invisalign; 2003.

[9] Wong BH (2002). Invisalign A, to Z. Am J Orthod Dentofacial Orthop.

[10] Eliades T, Eliades G, Watts DC (1999). Structural conformation of in vitro and in vivo aged orthodontic elastomeric modules. EurJ Orthod.

[11] Gracco A, Mazzoli A, Favoni O, Conti C, Ferraris P, Tosi G (2009). Short-term chemical and physical changes in Invisalign appliances. Aust Orthod J.

[12] Huget EF, Patrick KS, Nunez LJ (1990). Observations on the elastic behavior of a synthetic orthodontic elastomer. J Dent Res.

[13] Dupaix RB, Boyce MC (2005). Finite strain behavior of poly(ethylene terephthalate) (PET) and poly(ethylene terephthalate)-glycol (PETG). Polymer.

[14] Guarneri MP, Lombardo L, Gracco A, Siciliani G (2013). Lo stato dell’arte del trattamento con allineatori.

[15] Frick A, Rochman A (2004). Characterization of TPU-elastomers by thermal analysis (DSC). Polym Test.

[16] Lu QW, Macosko CW (2004). Comparing the compatibility of various functionalized polypropylenes with thermoplastic polyurethane (TPU). Polymer.

[17] Fang D, Zhang N, Chen H, Bai Y (2013). Dynamic stress relaxation of orthodontic thermoplastic materials in a simulated oral environment. Dent Mater J.

[18] Zhang N, Bai Y, Ding X, Zhang Y. Preparation and characterization of thermoplastic materials for invisible orthodontics. Dent Mater J. 2011. [Epub ahead of print] 10.4012/dmj.2011-120 22123023

[19] Watts DC, Cash AJ (1994). Analysis of optical transmission by 400–500 nm visible light into aesthetic dental biomaterials. J Dent.

[20] Martens H, Nielsen JP, Balling Engelsen S (2003). Light scattering and light absorbance separated by extended multiplicative signal correction. Application to near-infrared transmission analysis of powder mixtures. Anal Chem.

[21] Leininger RI, Hutson T, Jakobsen R (1987). Spectroscopic approaches to the investigation of interactions between artificial surfaces and protein. Ann N Y Acad Sci.

[22] Phua SK, Castillo E, Anderson JM, Hiltner A (1987). Biodegradation of a polyurethane in vitro. J Biomed Mater Res.

[23] Schollenberger CS, Stewart FD (1971). Thermoplastic polyurethane hydrolysis stability. J Elastoplast.

[24] Schuster S, Eliades G, Zinelis S, Eliades T, Bradley TG (2004). Structural conformation and leaching from in vitro aged and retrieved Invisalign appliances. Am J Orthod Dentofacial Orthop.

